# A compliance real-time monitoring system for the management of the brace usage in adolescent idiopathic scoliosis patients: a pilot study

**DOI:** 10.1186/s12891-021-03976-5

**Published:** 2021-02-05

**Authors:** Ce Zhu, Qiang Wu, Bing Xiao, Juehan Wang, Chao Luo, Quan Yu, Limin Liu, Yueming Song

**Affiliations:** 1 Department of Orthopedics Surgery and Orthopedics Research Institute, West China Hospital, Sichuan University, No. 37 Guoxue Road, Chengdu, 610041 Sichuan China; 2 Department of Spine Surgery, the Affiliated Hospital of Southwest Medical University, Luzhou, China; 3 Department of Rehabilitation Medicine, West China Hospital, Sichuan University, Chengdu, China; 4 Chengdu Spine Medical Technology Co., Ltd, Chengdu, China; 5 SiChuan NeoSource BioTektronics Limited, Chengdu, China

**Keywords:** Patient compliance, Real-time monitoring, Brace treatment, Adolescent idiopathic scoliosis

## Abstract

**Background:**

Patient compliance is essential to the effectiveness of brace treatment for adolescent idiopathic scoliosis (AIS) patients. Subjective measurements like questionnaires and inquiries proved to be arguably inaccurate. Although some scholars have applied temperature/force monitors to measuring patient compliance objectively, few studies to date could monitor patient compliance in real time. The objective of this study is to describe and evaluate a compliance real-time monitoring system of the brace usage in AIS patients.

**Methods:**

A compliance real-time monitoring system (specifically consisting of a compliance monitor, a WeChat Mini Program, a cloud-based storage system and a website backstage management system) was designed to manage the brace treatment. Thirty patients receiving brace treatment were enrolled. They were told to upload the data at least once a day. Clinicians downloaded the compliance data and communicated with the patients online based on their analysis of data at least once every 3 months. The measured force, quality compliance (measured force / baseline force), measured time, and quantity compliance (measured time/ prescribed time) were used to evaluate patient compliance. Patients were also asked to rate their satisfaction at the final follow-up.

**Results:**

Twenty-eight patients were included in the final analysis. The mean baseline force was 1.23 ± 0.28 N. The mean measured force was 0.79 ± 0.29 N. The mean quality compliance was 64.8 ± 22.2%. The prescribed time of all patients was 23 h. The mean measured time was 14.1 ± 2.9 h. The mean quantity compliance was 61.3 ± 12.6%. Both the quality and quantity compliance during the first 3 months of treatment was significantly lower than the latter 3 months. In this study, 96.4% (27/28) patients were satisfied with the use of the monitoring system, among whom 21.4% (6/28) are very satisfied and 75.0% (21/28) are somewhat satisfied.

**Conclusions:**

The compliance real-time monitoring system, without evaluating the clinical and radiographic outcomes for now, has already shown some feasibility and effectiveness for the management of the brace usage in AIS patients. This system, as a useful tool for online patient management and patient-clinician communication, would be potentially employed on a large scale in future for clinicians to improve the compliance and satisfaction of AIS patients who have received brace treatment.

## Background

Adolescent idiopathic scoliosis (AIS) is a three-dimensional deformity of the spine and it affects 1 ~ 3% of children between 10 and 16 years of age [1]. Brace treatment has been widely used for growing patients with curves larger than 25°, but smaller than 45° ~ 50°. And it has emerged as the only proven method of non-operative treatment [2].

The outcome of brace treatment depends on many factors such as age, gender, bone maturity, curve pattern, curve magnitude, and compliance [3]. Among them, compliance is a significant one for patients to achieve a satisfactory outcome of brace treatment. In order to better understand the role of compliance in brace treatment success, the comprehensiveness and accuracy of the measuring methods must be guaranteed.

Historically, patient compliance was measured subjectively by conducting questionnaires, reviewing a patient diary, or simply asking patients and their parents [4–8]. These subjective measurements were arguably inaccurate and could result in mistaken conclusions about brace treatment effectiveness [9]. Therefore, electronic devices were developed to measure medication compliance objectively. The electronic solution can be divided into three categories: temperature-based systems [2, 10–18], force-based systems [19–24], and force and temperature-combined systems [9]. A temperature-based system, with its temperature sensors, can record the brace wearing time (quantity) of the patients accurately, but cannot measure the wearing tightness (quality). In comparison, a force-based system or a force and temperature-combined system, with their respective monitors, can record both the wearing quantity and the wearing quality.

Some scholars have already applied compliance temperature/force monitors to the brace treatment of AIS patients in clinical practice [2, 9, 10, 13–18, 20–22]. The monitored data was firstly stored in the electronic device and then downloaded at the patients’ routine clinical visits (range 3 ~ 6 months). In this way, both clinicians and patients/parents could know how much time and how well the orthosis is worn after the analysis of the acquired data. However, the data only revealed the quantity and/or quality of brace usage during the past period before the patients returned to the clinic, so that patients were not able to make improvements in time. Therefore, it is better to find a way to measure patient compliance in real time, so as to improve AIS patients’ compliance with orthoses.

Mobile health (mHealth) is the practice of medical and public health supported by mobile devices such as phones, tablets, personal digital assistance, and wireless infrastructure [25]. The development of mHealth and popularization of smartphones enable patients to use mobile medical devices to collect their health data in real time, deliver and share the data with their doctors remotely, and receive feedback from the clinicians in time [26]. Some mHealth applications have been studied and applied to spine surgery patients in recent years [26–28]. However, to our knowledge, few studies had implemented an mHealth-based intervention to manage the brace usage in AIS patients. Hence, the objective of this study is to describe and evaluate a compliance real-time monitoring system of the brace usage in AIS patients.

## Methods

This study was approved by the ethics committee of West China Hospital of Sichuan University and informed consent was obtained from the patients and their parents. All methods were carried out in accordance with relevant guidelines and regulations.

### Compliance real-time monitoring system

**Fig. 1:**
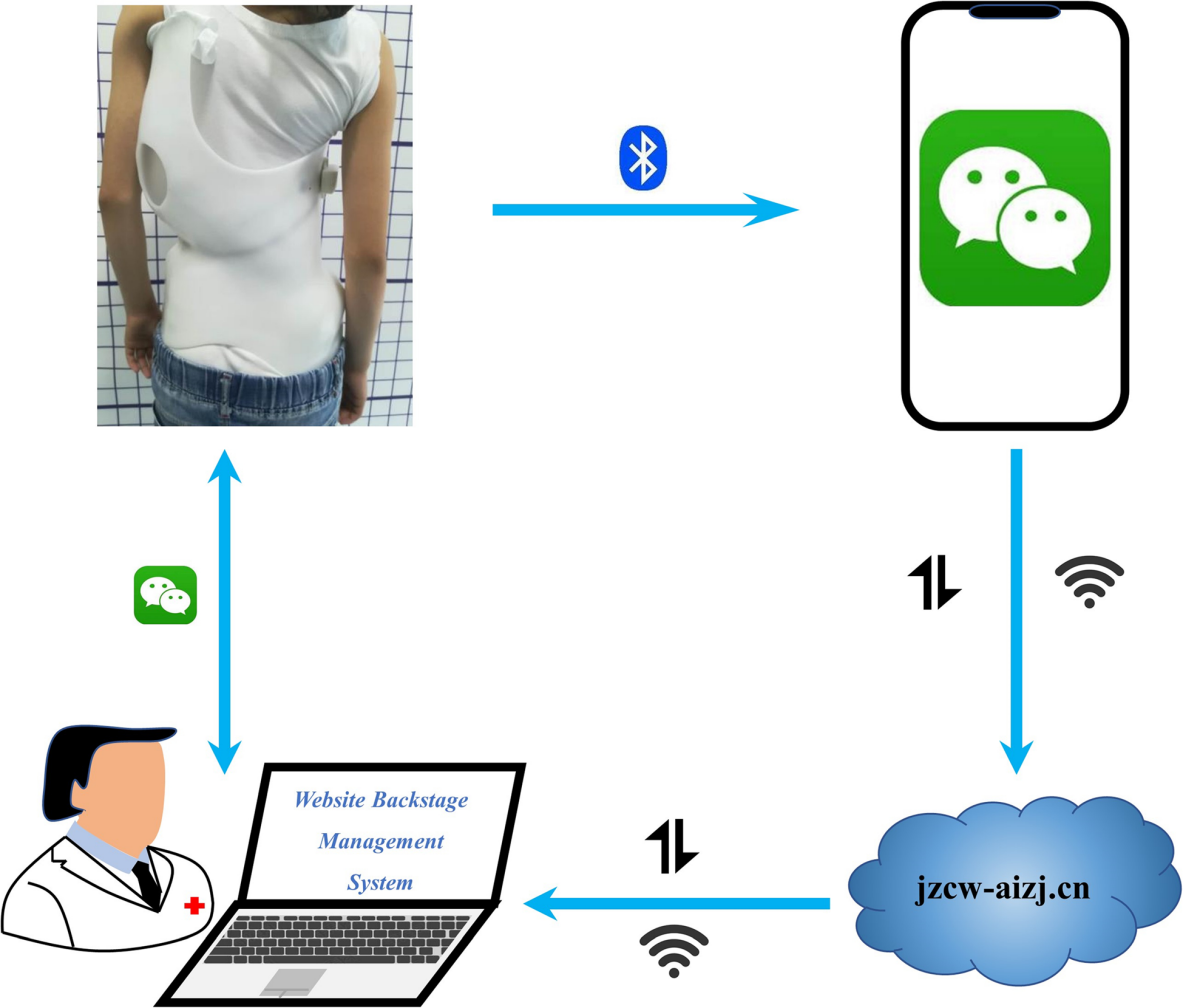
Schematic for the compliance real-time monitoring system which consists of a compliance monitor, a WeChat Mini Program, a cloud-based storage system, and a website backstage management system

**Fig. 2:**
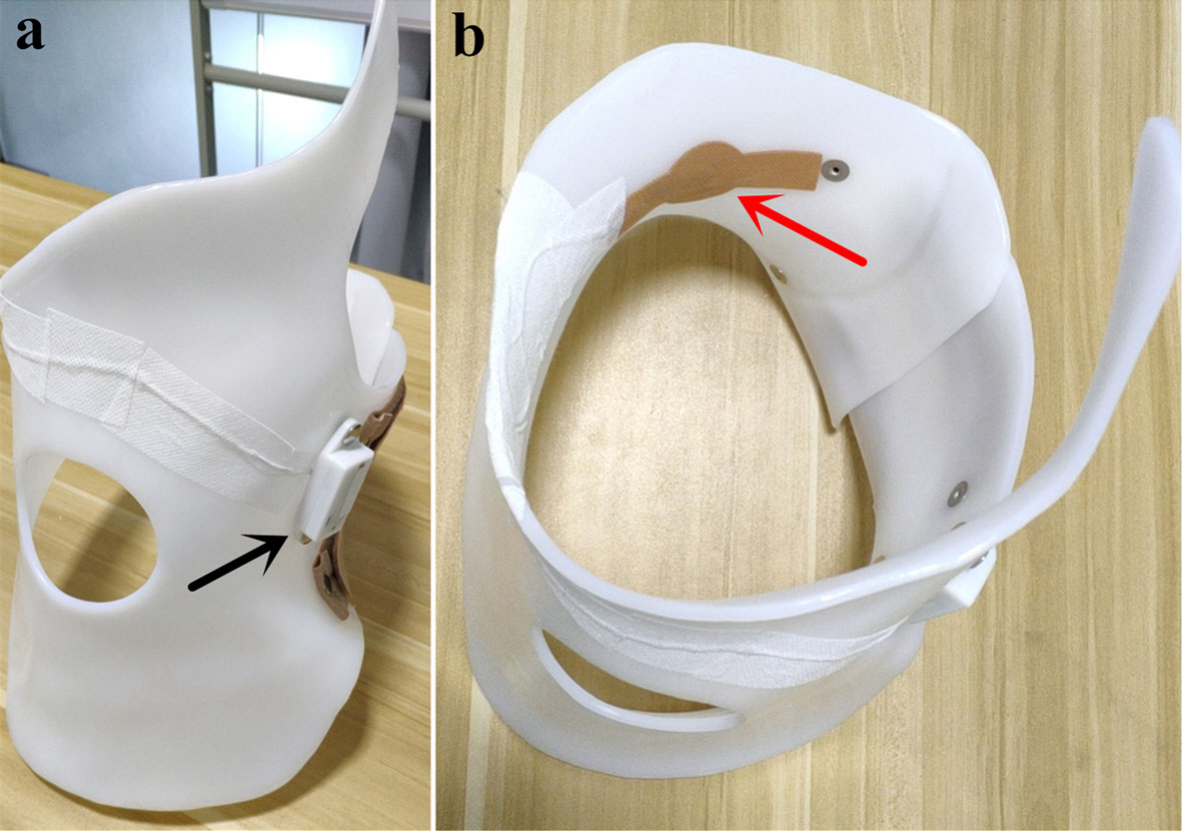
A compliance monitor embedded in a Chêneau brace: **(a)** the data logger (black arrow) was embedded in the outside surface of the brace; **(b)** the force sensor was attached to the inner surface with adhesive coverings at the major correction area without altering the corrective mechanism of the brace

**Fig. 3:**
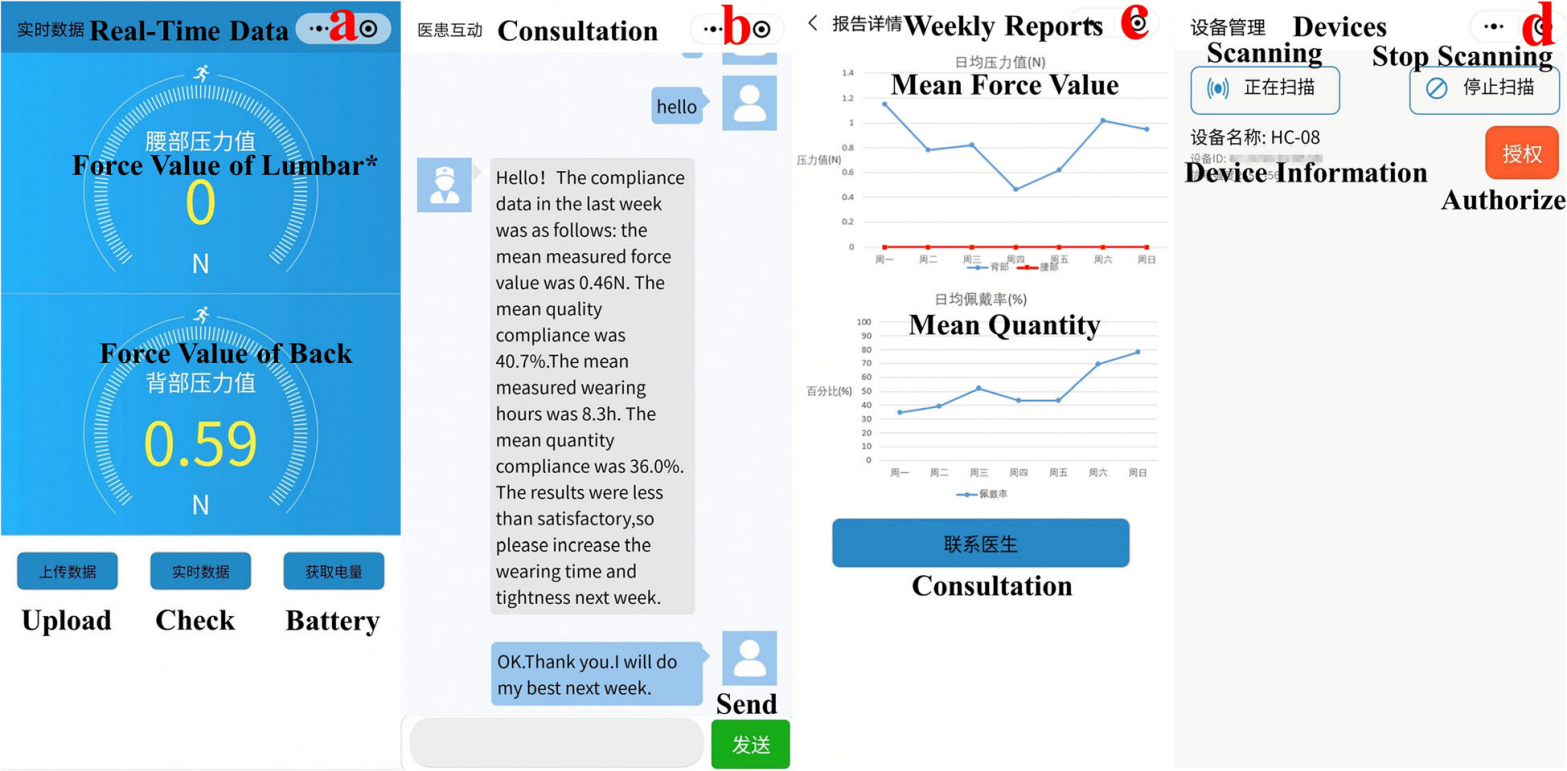
The user interface of the WeChat Mini Program: **(a)** Real-time Data Interface: patients or their parents can check and/or upload the compliance data; **(b)** Consultation Interface: patients or their parents can communicate with the clinicians; **(c)** Weekly Reports Interface: patients or their parents can review the compliance data of the last week; **(d)** Devices Interface: patients or their patients can bind their WeChat account with the compliance monitor. *Considering that we might add one more force sensor in the next iteration of the monitoring system, we presciently designed this program to support up to two force sensors (back and lumbar)

The components of the compliance real-time monitoring system (Fig. 1) include: a compliance monitor (Fig. 2), a WeChat Mini Program (Fig. 3), a cloud-based storage system, and a website backstage management system.

#### Compliance monitor

The compliance monitor consists of a battery-powered data logger (Fig. 2a) with a force sensor (Fig. 2b). The dimensions of the data logger are 75.2 mm × 28.7 mm × 16.0 mm. The device uses a lithium battery charged by USB and can last approximately 22.5 days on a single charge. The default sampling period is 0.5 min and at this sampling frequency, the monitor, with its large memory, could store 327.7 days’ force data. The force data can be transferred to the connected smart phone via wireless Bluetooth 4.0 communication.

The thin film force sensor (FSR-Model 400, Interlink Electronics, USA) is utilized to detect the force at the skin-brace interface. The sensing area of the sensor is 5.08 mm and its thickness is 0.3 mm. The maximum force that the transducer could measure is 20 N, which is suitable to this application based on other studies [21, 23].

#### WeChat mini program

WeChat Mini Program is an application of WeChat (Tencent, China) that can be used without downloading and installation. WeChat users can easily open the customized Mini Program by scanning its Quick response (QR) code or searching its name (*支具精灵, Zhi Ju Jing Ling*). Patients or their parents can access the WeChat app through their smartphones (Android or iOS) and bind their WeChat account with the compliance monitor (Fig. 3d). Subsequently, patients or their parents can check, review and/or upload the compliance data whenever they want (Fig. 3a and c). They can also communicate with the clinicians directly through the program (Fig. 3b).

#### Cloud-based storage system

Patients or their patients can upload the compliance data from the customized Mini Program on the smartphone to the cloud-based storage system at *jzcw-aizj.cn* by simply pressing a button within the Mini Program.

#### Website backstage management system

Clinicians can review or download the compliance data through the website backstage manage system and provide patients with necessary recommendations and counselling.

### System validation

This study enrolled the AIS patients who received brace treatment in West China Hospital to validate the compliance real-time monitoring system. The inclusion criteria were: (1) age ≥ 10 years or older at the time of brace treatment, (2) Risser 0 ~ 2, (3) curves 25° ~ 40°, and (4) no prior treatment [29].

Each of the enrolled patients was prescribed a Chêneau brace by the clinicians in our department. All braces were made by the same orthotist, who has more than 10 years’ experience. The data logger was embedded in the outside surface of the brace and the force sensor was attached to the inner surface with adhesive coverings at the major correction area, without altering the corrective mechanism of the brace. At initiation, patients and their parents were instructed in using the compliance real-time monitoring system. Then, clinicians measured patients’ baseline brace force and told them that the prescribed brace wearing time was 23 h per day. The present study used the same method to evaluate the quantity and quality of the brace usage as was adopted in the previous study by Lou et al. [21]. The quantity of brace usage was defined as measured time (force value>0) / prescribed time (23 h). The quality of brace usage was defined as measured force / baseline force.

The patients or their parents were requested to upload the data at least once a day to guarantee that the data stored in the cloud system were upgraded on a daily basis and without too much delay. Though it was favorable for the patients or their parents to upload the data much more often each day, most of the AIS patients could not do so, for in the daytime they might stay at school, where they were less likely to be allowed to use their smartphones when studying. Patients or their parents could consult the clinicians whenever they had any question about the treatment, and clinicians would reply to them timely after analyzing the patients’ compliance data. If patients didn’t contact their clinicians during the treatment, then clinicians would analyze the compliance data and communicated with the patients at least once every 3 months. When the quality compliance and/or quantity compliance of the patients were not satisfactory, clinicians would instruct patients or their parents online on what to do next. Suggestions included increasing the wearing time of the brace, adjusting the straps of the brace to the values that approximate the patient’s baseline force, etc.

Each patient used the system for 6 months. At the clinic visit in 6 months, patients were also asked to rate their satisfaction as being very satisfied, somewhat satisfied, somewhat dissatisfied, or very dissatisfied.

All data were analyzed through SPSS software (version 22.0; IBM Corp., Armonk, NY, USA). All values were presented as the mean ± standard deviation. Paired Student’s t-test was used to analyze the difference of measured force, quality compliance, measured time, and quantity compliance between the first and the latter 3 months of treatment. Statistical significance was set at *P* < 0.05.

## Results

A total of 30 AIS patients were enrolled in this study. Among them, there was one patient who found her data logger detached from the brace and broken, the other patient could not upload the data from her data logger to the smartphone. Therefore, 28 patients (5 males and 23 females) were included in the final analysis. The mean patient age was 12.4 ± 1.5 years (range: 10 ~ 15 years). The follow-up period of all patients was 6 months. The mean baseline brace force was 1.23 ± 0.28 N (range: 0.82 ~ 1.95 N). The mean measured force was 0.79 ± 0.29 N (range: 0.25 ~ 1.60 N). The mean quality compliance was 64.8 ± 22.2% (range: 29.2 ~ 127.7%). The prescribed time of all patients was 23 h. The mean measured time was 14.1 ± 2.9 h (range: 6.8 ~ 18.7 h). The mean quantity compliance was 61.3 ± 12.6% (range: 29.8 ~ 81.6%).

Details of measured force, quality compliance, measured time, and quantity compliance for each patient were listed in Table 1. The quality compliance during the first 3 months of treatment was significantly lower than the latter 3 months of treatment (49.1% vs. 80.5%, P<0.05). Similarly, the quantity compliance of the first 3 months was significantly less than that of the latter 3 months (52.3% vs. 70.3%, P<0.05).

**Table 1:** The quality and quantity of brace usage for the patients (*n* = 28)

Parameters	The First 3 Months	The Latter 3 Months	P
Baseline Force (N)	1.23 ± 0.28		/
Mesured Force (N)*	0.61 ± 0.20	0.98 ± 0.26	0.000
Quality Compliance (%)*	49.1 ± 10.4	80.5 ± 19.6	0.000
Prescribed Time (h)	23.0		/
Mesured Time (h)*	12.0 ± 2.4	16.1 ± 1.4	0.000
Quantity Compliance (%)*	52.3 ± 10.8	70.3 ± 6.4	0.000

*p < 0.05

In this study, 96.4% (27/28) patients are satisfied with the use of the monitoring system, among whom 21.4% (6/28) are very satisfied and 75.0% (21/28) are somewhat satisfied. There was only one patient who was somewhat dissatisfied with the system.

## Discussion

The measuring method of compliance is a key element in the accuracy of reported compliance. Previous studies used subjective ways such as questionnaires and diaries to measure the time that the brace has been worn during daily activities [4–8]. But the validity of such compliance data was questionable because patients may often overestimate their brace wearing time [10, 30]. With advances in technology, electronic devices were utilized to measure compliance. The most common of such devices were temperature and pressure/force sensors. The temperature sensors were proved to be effective to monitor wearing time [2, 10–18], but they could not measure the level of wearing tightness. Nicholson et al. [10] reported that the mean temperature at the skin-brace interface was 32.8 ± 1.6 °C. So, another limitation of the temperature sensors is that their accuracy may be affected when the ambient temperature reaches 30 ~ 40 °C level. In this study, the thin film force sensor was chosen to monitor the patient compliance. The primary advantage of this sensor is its ability to monitor wearing quantity and quality simultaneously. In addition, this small force sensor can be installed at any position of the brace surface and is easy to mount/dismount without destructing the corrective mechanism of the brace.

The influence of compliance is related to many factors. Takemitsu et al. [31] found that age correlated with compliance and younger patients showed higher compliance. Rahimi et al. [3] indicated that patient compliance might be improved if the orthotists would provide the opportunity for patients to choose the preferred appearance and construction. Nicholson et al. [10] considered that psychological feature could alter the amount of orthosis wearing hours. Daytime and nighttime wearing patterns are another two factors that could impact patient compliance, but which one is better is still controversial [2, 9, 10, 21, 23].

If these influential factors with brace treatment were considered, the patient compliance would be improved. Miller et al. [16] designed a randomized clinical trial to evaluate the direct effect of electronic monitoring (temperature probe) on brace-wearing compliance and found that patients, who were aware that their compliance was being monitored, would be more compliant than those who were unaware of such monitoring. A similar relation was found in the study by Karol et al. [15]. In this study, all patients and their parents were told at the beginning of the treatment that sensors embedded into patients’ brace would monitor their wearing time and wearing tightness of the brace. In addition, clinicians provided suggestions for patients/parents according to the actual downloaded compliance data at least once every 3 months since the time when the brace was prescribed. The mean measured time (14.1 ± 2.9 h) and the mean quality compliance (64.8 ± 22.2%) were greater than that in previous studies [16, 32]. The increased compliance seen in this study can be largely attributed to the timely and frequent communication between clinicians and patients/parents. With the aid of the compliance real-time monitoring system, patients or their parents could consult clinicians whenever they had any question about the treatment and receive the professional and individualized guidance from clinicians in time. Additionally, both the quality and quantity compliance during the first 3 months of treatment was significantly lower than the latter 3 months. For one thing, the first 3 months might be a transition period as the patients were getting accustomed to their new braces. For another, the feedback provided by clinicians based on the data of the first 3 months might amplify the Hawthorne effect [16] on these enrolled patients.

Patients and their parents’ attitudes towards electronic monitors are significant to the application and popularization of these monitors. Donzelli et al. [14] investigated the attitudes of 364 parents and patients with regards to the use of temperature sensors. They also found that the mean rate of parents stating a completely or at least partially positive attitude was 94.0% while it was 85.6% among patients. In the present study, the percentage of patients who were very satisfied or somewhat satisfied reaches 96.4%. Such a high satisfaction would be attributed to the following reasons more specifically: (1) patients and their parents could review the historical compliance data and real-time wearing tightness whenever they want, (2) patients and their patients could receive regular professional and individualized advice from clinicians according to the patients’ data, (3) patients or their parents could consult clinicians about the questions they met during the patient’s brace treatment without restrictions of time and place, and (4) minimize the need for in-person visits to the clinic, thus maximize the reduction of patients’ financial and time expense, especially for patients from remote areas.

The main strength of this study is the establishment of an integrated compliance real-time monitoring system that has proved to be efficient for the brace usage management in AIS patients. This system not only allows patients to perform active self-evaluation of their treatment, receive regular individualized guidance, and consult their clinicians online, but also help clinicians to make necessary adjustments timely or find better solutions for diagnosis and treatment. To date, the most commonly prescribed wearing time is 23 h per day, which is generally based on the past clinical experience of clinicians. The optimal wearing tightness of brace treatment is still unknown (being too tight can cause pain and pressure ulcers, while being too loose will compromise therapeutic efficacy). As data are accumulated over time through the compliance real-time monitoring system, it is believed that the ideal values of the wearing time and applied force will be worked out via cognitive computing, predictive analytics, artificial intelligence, and other advanced technologies.

Some limitations still exist. Since this pilot study mainly aimed to validate the feasibility and practicality of the system in question, it did not expect to see dramatic clinical changes in the brace treatment of AIS patients after the system was adopted. That is why only a small sample size of 30 patients was chosen and a short follow-up time of 6 months was investigated. However, this is not enough if the goal of the study is to provide a strong support for a large-scale application of the system in the future. In addition to the present study, at least three tasks will be fulfilled further: an analysis of the clinical and radiographic outcomes of patients who have received the treatment; necessary modifications and upgrading of the system after this initial test (to name a few, more force sensors will be added to improve the accuracy of the monitor, the data collected by the monitor can be uploaded to the cloud system synchronously without extra help from patients or their parents as long as the internet is connected, and the system will automatically send a reminder to patients or their parents on the ideal values of the wearing time and applied force that they can adjust to, based on large-scale computing); evaluations of the updated system with a larger number of patients, control groups and longer follow-up.

## Conclusions

The compliance real-time monitoring system, without evaluating the clinical and radiographic outcomes for now, has already shown some feasibility and effectiveness for the management of the brace usage in AIS patients. This system, as a useful tool for online patient management and patient-clinician communication, would be potentially employed on a large scale in future for clinicians to improve the compliance and satisfaction of AIS patients who have received the brace treatment.

## Data Availability

Data will be available upon request to the corresponding author.

## Abbreviations

AIS: Adolescent idiopathic scoliosis
mHealth: Mobile health
QR: Quick response
